# A case report of a lung abscess as a rare complication of a pyogenic liver abscess

**DOI:** 10.1097/MD.0000000000027789

**Published:** 2021-12-03

**Authors:** Hee Yeon Kim, Hyo-Jin Lee

**Affiliations:** Department of Internal Medicine, Uijeongbu St. Mary's Hospital, College of Medicine, The Catholic University of Korea, Uijeongbu-si, Gyeonggi-do, Republic of Korea.

**Keywords:** complication, hepatobronchial fistula, liver abscess, lung abscess, pyogenic

## Abstract

**Rationale:**

A hepatobronchial fistula and lung abscess following a pyogenic liver abscess is a rare entity and it is not easy to diagnose this condition based on the symptoms and chest radiography.

**Patient concerns:**

An 81-year-old man presented with productive cough and dyspnea.

**Diagnosis:**

Chest radiography indicated increased opacities in the right lower lung field with an air-fluid level suggestive of pneumonia complicated by a lung abscess. Chest and abdominal computed tomography revealed an abscess in the right lower lung field that bordered an abscess at segment 7 of the liver. Tubography confirmed a fistula between the liver and lung abscesses.

**Interventions:**

Due to communication between 2 abscesses, transhepatic approach was done instead of transpleural approach to avoid complications.

**Outcomes:**

A liver abscess complicated by a lung abscess was resolved following percutaneous transhepatic drainage of the liver abscess and antibiotic administration.

**Lessons:**

Though uncommon, the lack of suspicion of sub-diaphragmatic liver abscess often lead to a delay in diagnosis and proper treatment. Our case implies the importance of computed tomography in early diagnosis of liver abscess in case of lung abscess in the right lower lung field.

## 1 Introduction

Although several complications can follow pyogenic liver abscess, cases with hepatobronchial fistula and lung abscess secondary to liver abscess are rare. Presentation of hepatobronchial fistula includes bile-stained sputum, cough, dyspnea, and/or fever. Without the characteristic symptoms, it is difficult to diagnose hepatobronchial fistula and lung abscess complicating a pyogenic liver abscess based on initial presentation and simple radiography.^[1]^ Here, we discuss a case of liver abscess that penetrated into the thoracic cavity with formation of a lung abscess that initially was suspected of lung abscess as a complication of pneumonia.

## 2 Case report

An 81-year-old man visited our emergency department with a 3-day history of productive cough with yellowish sputum and dyspnea. He reported a 50-pack-year history of cigarette smoking and a medical history of hypertension but not of diabetes. The patient had undergone subtotal gastrectomy with a Billroth II anastomosis due to gastric ulcer perforation 40 years prior. Physical examination revealed tenderness in the right upper abdomen but no fever. The patient's pulse rate was 82 beats/min, and his respiratory rate was 20 breaths/min. Laboratory tests revealed a white blood cell count of 15,570/μL with 92.7% neutrophils, fasting glucose of 105 mg/d, aspartate aminotransferase of 34 U/L, alanine aminotransferase of 19 U/L, alkaline phosphatase of 298 U/L, and gamma-glutamyl transferase of 236 U/L. The C-reactive protein level was 25.31 mg/dL (normal range: <0.3 mg/dL), and the procalcitonin level was 13.1 ng/mL (normal range: <0.05 ng/mL). Serum antibody testing for amoeba species was negative.

**Figure 1:**
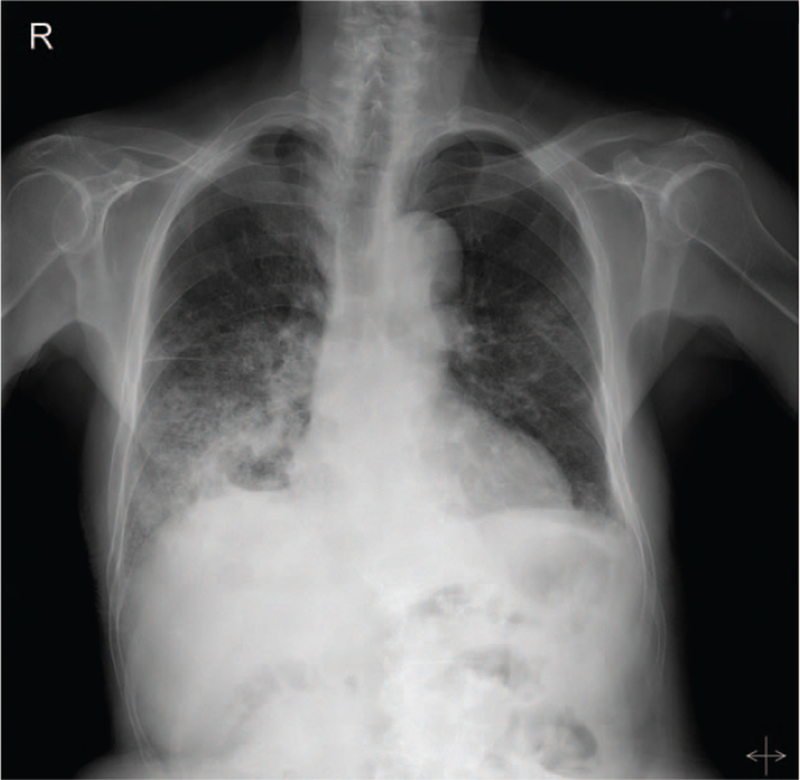
Chest radiography indicates increased opacities in the right lower lung field with an air-fluid level.

**Figure 2:**
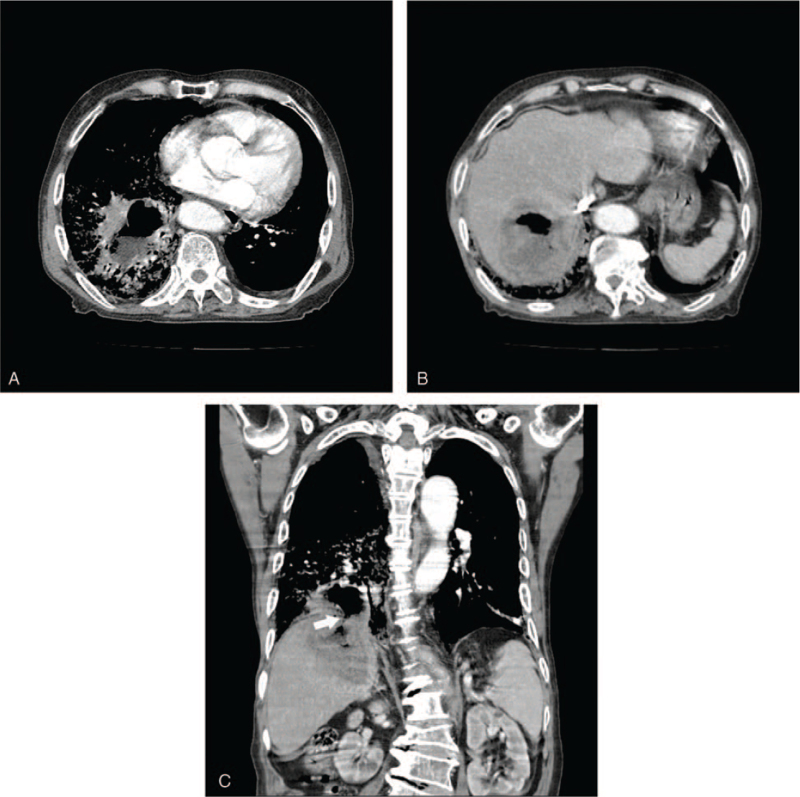
(A) A computed tomography (CT) scan of the chest depicts a 6.1-cm-sized air- and fluid-filled cavitary lesion with surrounding air space consolidation in the right lower lung field. (B) CT indicates a liver abscess with thick walls at segment 7 of the liver. (C) A fistula at the diaphragm between the 2 abscesses is visible in the coronal plane (arrow).

Chest radiography revealed increased opacities in the right lower lung field with an air-fluid level (Fig. 1). A computed tomography (CT) imaging of the chest indicated a 6.1 × 3.3-cm-sized air- and fluid-filled cavitary lesion with surrounding air space consolidation (Fig. 2A). This lesion bordered a 7.2 × 6.6-cm-sized thick-walled lesion with an air-fluid level suggestive of abscess formation in segment 7 (S7) of the liver (Fig. 2B). The coronal view of the CT scan revealed a defect in the diaphragm through which a communication between 2 cavities can occur (Fig. 2C). These findings suggest that the thoracic cavitary lesion was a lung abscess secondary to invasion of the liver abscess into the thoracic cavity.

**Figure 3:**
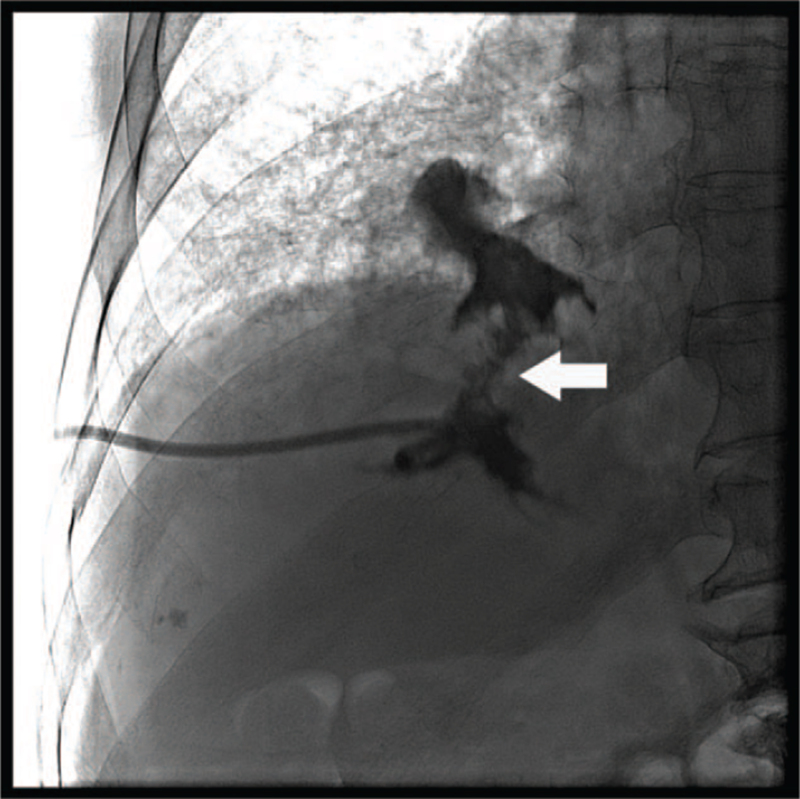
Tubography through a pigtail catheter reveals contrast media leakage via a defect in the diaphragm between the liver and lung (arrow).

Ultrasonography-guided percutaneous drainage of the liver abscess was performed. On tubography conducted through a pigtail catheter, communication between the liver and lung was visualized (Fig. 3). Therefore, the diagnosis of hepatobronchial fistula was confirmed on the basis of tubography findings. A total of 367 mL of yellowish brown pus was drained. Cultures from the drained material identified *Escherichia coli*-producing extended-spectrum beta-lactamases. Sputum culture also revealed extended-spectrum beta-lactamase-producing *E coli*. Blood cultures were sterile. Initially, the patient received cefoperazone 2 g/d and sulbactam 2 g/d intravenously combined with metronidazole 1500 mg/d intravenously for 5 days. The antibiotic was changed to piperacillin 12 g/d and tazobactam 1.5 g/d based on testing of bacterial susceptibility to antibiotics. The piperacillin/tazobactam was administered for 15 days. On day 15, the catheter was removed due to reduced fluid drainage. After discharge, amoxicillin 1500 mg/d and clavulanate 375 mg/d were administered orally for 21 consecutive days. A CT scan obtained 6 weeks after diagnosis indicated regression of the abscess pocket in S7 of the liver and in the right lower lung.

## 3 Discussion

Liver abscesses that arise close to the diaphragm can penetrate into the thoracic cavity, causing acquired hepatobronchial fistula and lung abscess, especially in amebic disease of the liver.^[2,3]^ In previous studies, hepatobronchial fistula has presented with bile-stained sputum, cough, dyspnea, and/or fever.^[1]^ Diagnosis of hepatobronchial fistula following a liver abscess generally is based on CT studies. An air- and fluid-filled liver abscess cavity located just below the diaphragm in continuity with a lung abscess in the right lower lung field on CT scan indicates the presence of hepatobronchial fistula. Percutaneous tubography can delineate a hepatobronchial fistula after injection of contrast media.^[4]^

Our patient initially was suspected of having pneumonia complicated by a lung abscess based on the symptoms and chest radiography. However, CT scan revealed a liver abscess edged with a lung abscess, which communicated through a hepatobronchial fistula. Without the characteristic symptoms of a productive cough with bile-stained sputum in addition to fever and leukocytosis, it is not easy to diagnose this condition prior to CT scan. Therefore, the possibility of liver abscess complicated by lung abscess should be considered in patients with a lung abscess in the right lower lung field.

To date, there is lack of definitive guidelines for management of lung abscesses that develop following penetrating liver abscess. Therapeutic strategies range from minimally invasive methods to aggressive surgery.^[5]^ In our case, percutaneous transpleural drainage was deferred due to the potential risk of hemothorax, pneumothorax, pyothorax, and bronchopleural fistula formation.^[6]^ In a case of a pure lung abscess, percutaneous transpleural drainage is the only approach despite the risk of complications. However, our case suggests that a lung abscess caused by a penetrating liver abscess can be drained successfully using a drainage catheter inserted into the liver abscess in the presence of communication between the lung and liver abscesses. A previous study reported that a lung abscess caused by a penetrating liver abscess was treated successfully by percutaneous transhepatic drainage through the diaphragmatic fistula. However, this technique is difficult to approach the diaphragmatic fistula and poses a higher risk of complications.^[7]^

In summary, hepatobronchial fistula and lung abscess are rare complications of liver abscess. Clinicians should be aware of the possibility of hepatobronchial fistula in patients with a lung abscess in the right lower lung field that also borders a liver abscess. Early computed tomography imaging should be considered to avoid a delay in diagnosis and apply appropriate treatments timely. Unlike a pure lung abscess, lung abscess caused by a penetrating liver abscess can be treated with percutaneous transhepatic drainage of the liver abscess and antibiotic administration when communication between the 2 abscesses is confirmed.

### 3.1 Informed consent

This case report was approved by the Ethics Committee of Uijeongbu St. Mary's Hospital. Written informed consent for the publication of this case report was waived because of the use of entirely anonymized images from which the individual cannot be identified.

## Author contributions

**Data curation:** Hee Yeon Kim.

**Supervision:** Hyo-Jin Lee.

**Writing – original draft:** Hee Yeon Kim.

**Writing – review & editing:** Hyo-Jin Lee.

## References

[1] Liao GQ Wang H Zhu GY. Management of acquired bronchobiliary fistula: a systematic literature review of 68 cases published in 30 years. World J Gastroenterol 2011;17:3842–9. 21987628 10.3748/wjg.v17.i33.3842 PMC3181447

[2] Varela Vega M Durán F Geribaldi N. Hepatobronchial fistula: a rare complication of liver abscess. Cir Esp 2017;95:410–1. 28041687 10.1016/j.ciresp.2016.10.012

[3] Meng XY Wu JX. Perforated amebic liver abscess: clinical analysis of 110 cases. South Med J 1994;87:985–90. 7939926 10.1097/00007611-199410000-00004

[4] Yoon DH Shim JH Lee WJ. Percutaneous management of a bronchobiliary fistula after radiofrequency ablation in a patient with hepatocellular carcinoma. Korean J Radiol 2009;10:411–5. 19568472 10.3348/kjr.2009.10.4.411 PMC2702053

[5] Kontoravdis N Panagiotopoulos N Lawrence D. The challenging management of hepatopulmonary fistulas. J Thorac Dis 2014;6:1336–9. 25276379 10.3978/j.issn.2072-1439.2014.07.19 PMC4178102

[6] Suh JH Park CB. Bronchopleurobiliary fistula following right lower lobectomy in a patient with prior hepatic abscess: a case report. Ann Transl Med 2020;8:1464 doi: 10.21037/atm-20-2776. 33313209 10.21037/atm-20-2776 PMC7723620

[7] Taniguchi M Morita S Ueno E. Percutaneous transhepatic drainage of lung abscess through a diaphragmatic fistula caused by a penetrating liver abscess. Jpn J Radiol 2011;29:663–6. 21956374 10.1007/s11604-011-0605-7

